# Effectiveness of the Shenzhuo formula in the treatment of patients with macroalbuminuria secondary to diabetic kidney disease: protocol update and statistical analysis plan

**DOI:** 10.1186/s13063-021-05961-8

**Published:** 2022-01-20

**Authors:** Yu Wei, Yi-Shan Huang, Ze Yang, Xinmiao Wang, Yanbo Li, Ying Zhang, Lin-Hua Zhao, Xiaolin Tong

**Affiliations:** 1 Department of Endocrinology, Guang’anmen Hospital, China Academy of Chinese Medical Sciences, Beijing, 100053 China; 2 Graduate College, Beijing University of Traditional Chinese Medicine, Beijing, 100029 China; 3 Post-doctoral research stations, China Academy of Chinese Medical Sciences, Beijing, 100010 China; 4 School of Traditional Chinese Medicine, Beijing University of Traditional Chinese Medicine, Beijing, 100029 China; 5 Department of Endocrinology, Affiliated Hospital to Changchun University of Chinese Medicine, Changchun, 130000 China

**Keywords:** Statistical analysis plan, Diabetic kidney disease, Shenzhuo Formula, Irbesartan, Clinical effectiveness

## Abstract

**Background:**

Diabetic kidney disease (DKD) is a significant complication of diabetes and has garnered considerable attention. Our previous retrospective study indicated that Shenzhuo formula (SZF) potentially reduces macroalbuminuria secondary to DKD.

**Methods:**

This trial is a 24-week, randomized, multicentric, double-blinded, double-dummy clinical trial. A total of 120 patients with DKD will be equally and randomly divided into two groups: SZF+ irbesartan simulator or irbesartan + SZF simulator. The 24-h urinary protein change from baseline to week 24 is the primary outcome measure. The secondary outcome measures include serum creatinine, estimated glomerular filtration rate, urinary albumin excretion rate, improvement in traditional Chinese medicine symptoms, fasting blood glucose, 2-h postprandial plasma glucose, hemoglobin A1c, cholesterol, triglycerides, high density lipoprotein, low density lipoprotein, blood pressure, albumin to creatinine ratio, and the Audit of Diabetes-Dependent Quality of Life 19. Our recruitment began in May 2015; currently, we have recruited 100 participants, with a designed maximum sample size of 120. The interim results were reviewed at *N* = 60, and continuing recruitment was recommended. This statistical analysis plan includes our approach to missing data imputation, primary and secondary outcomes analyses, and safety endpoints.

**Discussion:**

This statistical analysis plan will standardize the clinical trial’s statistical analysis and avoid outcome selective reporting bias and data-driven analysis. This trial will provide further clinical evidence regarding the effectiveness of SZF in managing macroalbuminuria secondary to DKD.

**Trial registration:**

Chinese Clinical Trial Registry ChiCTR-ICR-15006311. Registered on 26 May 2013. http://www.chictr.org.cn/showproj.aspx?proj=10862

**Supplementary Information:**

The online version contains supplementary material available at 10.1186/s13063-021-05961-8.

## Background

Diabetic kidney disease (DKD) is a leading cause of chronic kidney disease (CKD) in developed countries and is rapidly becoming the leading cause in developing countries as well [1]. It is one of the most common complications of diabetes mellitus affecting approximately 20–40% of diabetic patients in China; furthermore, it has replaced chronic glomerulonephritis as the leading cause of end-stage renal disease in middle-aged and elderly patients [2–4]. To address this, renin-angiotensin-aldosterone system (RAAS) inhibitors have recently been used; however, the prevalence of DKD remains unchanged [5, 6]. Therefore, exploring effective drugs for preventing DKD occurrence and development is critical.

Recently, increasing extensive clinical experience has supported the use of traditional Chinese medicine (TCM) for DKD. TCM with angiotensin receptor inhibitor treatment can reduce proteinuria in patients with type 2 diabetes [7]. Our previous retrospective study found that the Shenzhuo formula (SZF) improves glomerular filtration rate (GFR) and reduces 24-h urinary protein (24 h-UP) [8]. Another study of 88 patients with DKD found that the SZF improved estimated glomerular filtration rate (eGFR) and slowed DKD progression [8].

A previous study evaluated the 2-year effectiveness of SZF in the treatment of DKD and showed similar results with no significant changes in liver function and routine blood test results compared with the baseline; additionally, no patient experienced discomfort or severe adverse events [9].

However, comparisons between the effectiveness of SZF and RAAS inhibitors in patients with DKD remain lacking. Therefore, we designed and implemented a trial to investigate the effectiveness and safety of SZF and irbesartan in treating patients with DKD and macroalbuminuria. We hypothesized that SZF alone can improve macroalbuminuria secondary to DKD. Using SZF, we conducted a 24-week, randomized, multicentric, double-blinded, double-dummy, controlled clinical trial. The research protocol was previously reported [10]. The original protocol provides more details regarding the trial rationale, eligibility criteria, interventions, concomitant treatments, study visits, and data management.

Here, we describe the study’s pre-determined statistical analysis plan (SAP). To prevent reporting bias and data-driven interpretation, the SAP will be applied at the termination of the trial. The SAP (version 2.0 20180626) was completed before the data analysis, and the investigators adhered to the requirements in data analysis, according to “Guidelines for the Content of Statistical Analysis Plans in Clinical Trials” published in JAMA [11] (see Additional file 1 populated checklist). It includes our approach to imputing missing data and analyzing primary and secondary outcomes; additionally, safety and baseline analysis was conducted, and total adverse effects were analyzed.

## Study methods

### Trial design

**Fig. 1:**
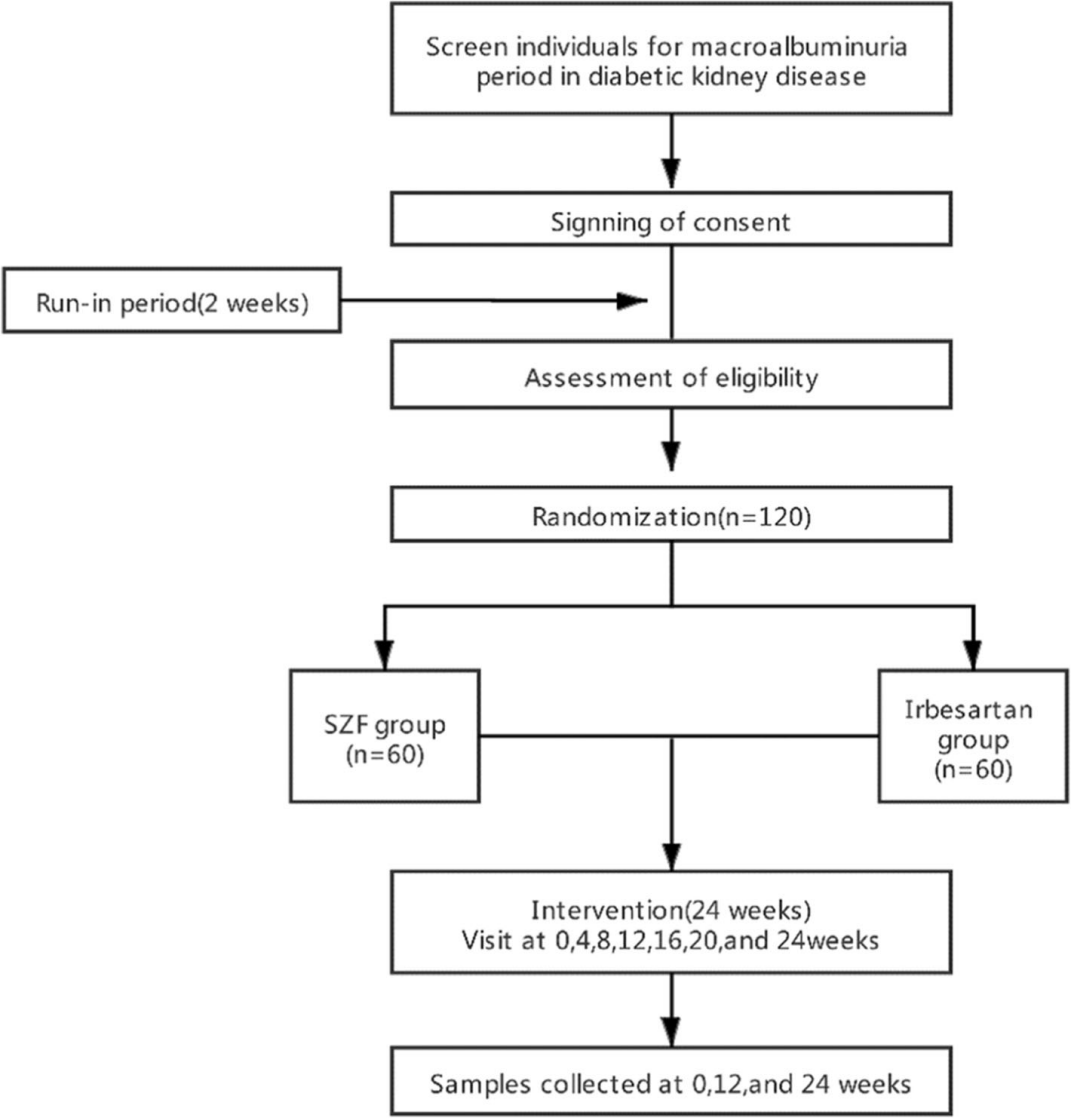
Flow diagram of the study

The SZF clinical trial will be conducted in 11 centers and will recruit 120 patients with macroalbuminuria secondary to DKD. In the protocol, we specified 120 subjects from nine hospitals will be included. However, three centers have been deleted because they have not carried out patient recruitment; in addition, we added five centers to hasten the trial. These hospitals include The Second Affiliated Hospital of Shaanxi University of TCM, Xianyang; Zhengzhou Hospital of TCM, Zhengzhou; Hangzhou Hospital of TCM, Hangzhou; The Affiliated Hospital to Changchun University of Chinese Medicine, Changchun; and Zhejiang Hospital of TCM, Hangzhou. The newly added centers have passed the ethical review by the Ethics Committee of Guang’anmen Hospital. To ensure quality, all investigators will receive intensive training regarding the conduct of the research and intervention before recruitment. The flow diagram shown in Fig. 1 provides a brief description of the research process. Our previous protocol defines the sample size calculation [10].

### Randomization and blinding

Eligible patients will be randomized in a 1:1 allocation ratio using a central permuted block randomization procedure (block size 4) with block sizes. The groups will include the TCM group, which will receive SZF and stimulated irbesartan, and the Western medicine group, which will receive SZF simulation agents and irbesartan capsules. Using the PROC PLAN process statement of SAS 9.4 (SAS/STAT Software 9.4, SAS Institute, Inc., Cary, NC, USA), a random arrangement (random coding table) of 120 subjects will be generated according to the number of seeds. The Institute of Basic Research in Clinical Medicine of the China Academy of Chinese Medical Sciences provides the random arrangement, 24-h emergency code break, and medical information. Participants, investigators, and statisticians will be blinded to treatment assignments.

SZF drugs and SZF simulators have identical shape, smell, and color, which were consistent when dissolved in water. Irbesartan capsules and simulators had consistent scent and appearance when the capsule was opened.

### Interim analysis

Independent biostatisticians will perform interim analysis once 60 patients complete the 24-week treatment, which is 50% of the planned number of patients. The primary and secondary outcomes and safety assessments will be evaluated to compare the effects and safety of SZF and irbesartan. The Data Monitoring Committee would recommend continuing, discontinuing, or modifying the trial, if concerns regarding the effect and safety of the participants will arise.

A recommendation of trial termination would be considered by the data management, clinical trial institutions, ethics committees, and sponsors if achieving the difference in treatment effects is unlikely or if intolerable adverse effects occurred. The detailed results remain confidential to the investigators. The interim analysis was conducted in September 2018, and continued recruitment was recommended.

### Criteria for trial discontinuation

Trial abortion refers to premature discontinuation of the clinical trial. The purpose of trial abortion is to protect subject equity, guarantee trial quality, and avoid unnecessary economic losses. The trial will be terminated if any of the following situations occur:

(1) Serious safety issues occurring during the trial warrant prompt discontinuation.

(2) Drug treatments found to be significantly inadequate or even ineffective in the trial to be of clinical value should be suspended, to avoid patient treatment delays and economic losses.

(3) The clinical trial protocol is found to have a significant lapse or a well-designed protocol that has undergone substantial deviations in implementation, increasing the difficulty in evaluating the drug effect.

(4) The sponsor requests discontinuation (e.g., reasons for funding, administration, etc.).

(5) Withdrawal of the trial by the administration.

## Statistical principles

### Overall principles

After completion of the trial, all the statistical analysis tests will use two-sided tests with a nominal alpha level of 0.05, using the SAS [10, 12]. All confidence interval (CI) reports will be 95% CIs. For each outcome, the null hypothesis is the absence of difference between the intervention groups.

The description of measurement indicators will calculate the mean, standard deviation, median, minimum, maximum, and interquartile range (Q3–Q1). Normal continuous variables will be presented as the mean and standard deviation; non-normally distributed continuous variables will be presented as median with interquartile range. Counting indicators will be presented as the frequency and percentage of each category.

Inter-group comparisons will be performed with independent two-sample *t*-tests (normality, homogeneity of variance) or Wilcoxon rank-sum test (non-normality, heterogeneity of variance). Comparisons within normal and nonnormally distributed groups will be performed using paired *t*-test and paired rank-sum test, respectively. *χ*^2^ test or Fisher’s exact probability will be needed for count metrics, and the Wilcoxon rank-sum test will be used for categorical variables. A generalized linear mixed model analysis will be performed on the repeated measured outcome.

In situations where an imbalance in the baseline indicators exists, an adjustment will be required for the efficacy comparison of variables, such as age, sex, and concomitant treatment. The covariance method will be used for continuous variables, and logistic regression analysis was used for count metrics and hierarchical metrics.

### Definition of analysis sets

#### Full analysis set

Patients who have received treatment at least once and meet the primary objective after treatment will be included in the fully analysis set (FAS).

#### Per protocol set

The subjects who were selected in the FAS and received at least 50% of scheduled treatment with no major protocol violations will be included in the per protocol set (PPS). The PPS will be determined prior to the unblinding of the data for the final analysis and will include the following:

All visits planned and the main data completed.

The inclusion satisfied and the exclusion inapplicable.

No significant violations of the trial protocol occurred (described in adherence and protocol deviations).

#### Safety set

The safety analysis will analyze all the patients who used the intervention drug at least once and underwent one safety assessment.

### Adherence and protocol deviations

Adherence is determined by the participants at each visit and according to medication adherence. We will describe the proportion of participants using frequency distributions for both groups. Medication compliance will be calculated and compared between two groups according to the ratio of actual medicine administered and the ideal dose that should be received. The medicine distributed to the patients and the medicine not administered will be recorded every 4 weeks.

Significant protocol violations are those that potentially affect the primary measures, such as receiving protocol prohibited medicine, increasing the safety risk of the participants, and ethical concerns.

## Trial population

### Eligibility criteria

This study includes patients with DKD who had macroalbuminuria, and controlled serum creatinine, blood pressure, and hemoglobin A1c levels (HbA1c). Patients with type 1 diabetes or those who received potassium-sparing diuretics are excluded from the study.

### Case enrollment and completion

The enrollment and the number of completed cases will be summarized for each center. The numbers of enrolled cases and each analyzed dataset will be described. Participants may withdraw from the trial intervention and/or fail to provide follow-up data. In addition, participants may withdraw their consent. We will report these items (Table 1), and the total dropout rate will be compared between the two groups.

**Table 1:** Withdrawals

Num	Withdrew at…	Reason to withdraw	Type of withdrawal
1	xx	xx	e.g., withdrew consent
2	xx	xx	e.g., stopped intervention early, provided data at follow-up
…	xx	xx	e.g., stopped intervention early, lost to follow-up
…	xx	xx	e.g., completed intervention, lost to follow-up
*n*	xx	xx	xx

### Baseline characteristics

The baseline characteristics of all participants in both groups will be shown in Table 2. The table will describe the following variables: age, height, weight, BMI, female sex, nationality, heart rate, systolic blood pressure, diastolic blood pressure, mean arterial pressure, respiration rate, drug or constitution allergic history, comorbid conditions, and concomitant treatment. The sample will be described according to the full analysis set. The baseline characteristics will be compared between groups.

**Table 2:** Baseline characteristics of participants prior to treatment

Characteristic	SZF, number, central tendency (variability)	Irbesartan, number, central tendency (variability)	All participants, number, central tendency (variability)	***p*** value
	*n* = xx	*n* = xx	*n* = xx	
**Demographics**				
Age (years)^a^	xx (xx)	xx (xx)	xx (xx)	.xx
Height (m)^a^	xx (xx)	xx (xx)	xx (xx)	.xx
Weight (kg)^a^	xx (xx)	xx (xx)	xx (xx)	.xx
BMI (kg/m^2^)^a^	xx (xx)	xx (xx)	xx (xx)	.xx
Female sex (*n*,%)^b^	xx (xx%)	xx (xx%)	xx (xx%)	.xx
Nationality (China, other)^b^	xx (xx%)	xx (xx%)	xx (xx%)	.xx
**Vital signs**				
Heart rate (beats per minute)^a^	xx (xx)	xx (xx)	xx (xx)	.xx
Systolic blood pressure (mmHg)^a^	xx (xx)	xx (xx)	xx (xx)	.xx
Diastolic blood pressure (mmHg)^a^	xx (xx)	xx (xx)	xx (xx)	.xx
Mean arterial pressure (mmHg)^a^	xx (xx)	xx (xx)	xx (xx)	.xx
Respiration rate(breath/min)^a^	xx (xx)	xx (xx)	xx (xx)	.xx
**Drug or constitution allergic history (yes or no)**^**b**^	xx (xx%)	xx (xx%)	xx (xx%)	.xx
**Comorbid conditions (*n*%)**				
Diabetic retinopathy^b^	xx (xx%)	xx (xx%)	xx (xx%)	.xx
…				
**Concomitant treatment**				
…^b^	xx(xx%)	xx(xx%)	xx(xx%)	.xx

^a^Number, mean, standard deviation

^b^Number, percentage

## Analysis methods

### Outcome measure analyses

#### Primary outcome analyses

The primary analysis will be conducted in the FAS and PPS. The change in 24-h UP (mg/24 h) is the primary outcome. The distribution and changes of 24-h UP at weeks 0, 4, 8, 12, 16, 20, and 24 of medication will be described. Intra- and inter-group comparisons will be made. The distributions of qualitative judgments of 24-h UP at weeks 4, 8, 12, 16, 20, and 24 will be described and compared between groups. The baseline 24-h UP is essential to assess the progression of UP; therefore, analysis of covariance (ANCOVA) will be performed to analyze the 24-h UP change from baseline to week 24, using baseline 24-h UP as a covariate. Interactions between site and group will also be tested.

Table 3 provides a reference for the qualitative determination of the variation. The efficiency rate will be calculated by summing the rate of clinical control, obvious therapeutic effect, and therapeutic effect (Table 4).

**Table 3:** 24-h UP qualitative judgments classification standard

Qualitative judgment	Standard
Clinical control	24-h UP< 0.5 g
Obvious therapeutic effect	24-h UP reduction ≥ 50%
With therapeutic effect	24-h UP reduction ≥ 20%
Invalid	Fail to reach the standards above

**Table 4:** Analysis of primary outcome

Time point	SZF, number, mean (SD)	Irbesartan, number, mean (SD)	Mean difference (95% CI)	***p*** value
Visit at	*n* = xx	*n* = xx		
Week 0	xx (xx)	xx (xx)	xx (xx to xx)	.xx
Week 4 ^a^	xx (xx)	xx (xx)	xx (xx to xx)	.xx
Week 8	xx (xx)	xx (xx)	xx (xx to xx)	.xx
Week 12	xx (xx)	xx (xx)	xx (xx to xx)	.xx
Week 16	xx (xx)	xx (xx)	xx (xx to xx)	.xx
Week 20	xx (xx)	xx (xx)	xx (xx to xx)	.xx
Week 24	xx (xx)	xx (xx)	xx (xx to xx)	.xx
Overall SZF effect^b^				.xx

^a,b^*p* values comparing between group differences at visit points post-randomization (a) and over the entire24-week trial (b: primary outcome)

#### Secondary outcome analyses

The secondary outcome of the FAS and PPS will be analyzed, including serum creatinine (SCr, umol/L), eGFR (mL/(min×1.73 m^2^) using CKD-EPI creatinine equation [13]), urinary albumin excretion rate (μg/min), improvement in TCM symptoms, fasting blood glucose (mmol/L), 2-h postprandial plasma glucose (mmol/L), HbA1C (%), cholesterol (mmol/L), triglycerides (mmol/L), high density lipoproteins (mmol/L), low density lipoproteins (mmol/L), blood pressure (mmHg), albumin to creatinine ratio (ACR, mg/g), and the audit of diabetes-dependent quality of life 19 (ADDQoL) [14] The distribution and changes of all the secondary outcomes at the baseline and at each visit point will be compared between and within groups (Table 5).

**Table 5:** Secondary outcome analysis

Time point	SZF, number, mean (SD)	Irbesartan, number, mean (SD)	Mean difference (95% CI)	***p*** Value
Scr at	*n* = xx	*n* = xx		
Week 0	xx (xx)	xx (xx)	xx (xx to xx)	.xx
Week 4	xx (xx)	xx (xx)	xx (xx to xx)	.xx
Week 8	xx (xx)	xx (xx)	xx (xx to xx)	.xx
Week 12	xx (xx)	xx (xx)	xx (xx to xx)	.xx
Week 16	xx (xx)	xx (xx)	xx (xx to xx)	.xx
Week 20	xx (xx)	xx (xx)	xx (xx to xx)	.xx
Week 24	xx (xx)	xx (xx)	xx (xx to xx)	.xx
Overall SZF effect ^a^				.xx
eGFR at	*n* = xx	*n* = xx		
Week 0	xx (xx)	xx (xx)	xx (xx to xx)	.xx
Week 4	xx (xx)	xx (xx)	xx (xx to xx)	.xx
Week 8	xx (xx)	xx (xx)	xx (xx to xx)	.xx
Week 12	xx (xx)	xx (xx)	xx (xx to xx)	.xx
Week 16	xx (xx)	xx (xx)	xx (xx to xx)	.xx
Week 20	xx (xx)	xx (xx)	xx (xx to xx)	.xx
Week 24	xx (xx)	xx (xx)	xx (xx to xx)	.xx
Overall SZF effect				.xx
UAER at	*n* = xx	*n* = xx		
Week 0	xx (xx)	xx (xx)	xx (xx to xx)	.xx
Week 4	xx (xx)	xx (xx)	xx (xx to xx)	.xx
Week 8	xx (xx)	xx (xx)	xx (xx to xx)	.xx
Week 12	xx (xx)	xx (xx)	xx (xx to xx)	.xx
Week 16	xx (xx)	xx (xx)	xx (xx to xx)	.xx
Week 20	xx (xx)	xx (xx)	xx (xx to xx)	.xx
Week 24	xx (xx)	xx (xx)	xx (xx to xx)	.xx
Overall SZF effect				.xx
FBG	*n* = xx	*n* = xx		
Week 0	xx (xx)	xx (xx)	xx (xx to xx)	.xx
Week 4	xx (xx)	xx (xx)	xx (xx to xx)	.xx
Week 8	xx (xx)	xx (xx)	xx (xx to xx)	.xx
Week 12	xx (xx)	xx (xx)	xx (xx to xx)	.xx
Week 16	xx (xx)	xx (xx)	xx (xx to xx)	.xx
Week 20	xx (xx)	xx (xx)	xx (xx to xx)	.xx
Week 24	xx (xx)	xx (xx)	xx (xx to xx)	.xx
Overall SZF effect				.xx
2hPG at	*n* = xx	*n* = xx		
Week 0	xx (xx)	xx (xx)	xx (xx to xx)	.xx
Week 4	xx (xx)	xx (xx)	xx (xx to xx)	.xx
Week 8	xx (xx)	xx (xx)	xx (xx to xx)	.xx
Week 12	xx (xx)	xx (xx)	xx (xx to xx)	.xx
Week 16	xx (xx)	xx (xx)	xx (xx to xx)	.xx
Week 20	xx (xx)	xx (xx)	xx (xx to xx)	.xx
Week 24	xx (xx)	xx (xx)	xx (xx to xx)	.xx
Overall SZF effect				.xx
TC at	*n* = xx	*n* = xx		
Week 0	xx (xx)	xx (xx)	xx (xx to xx)	.xx
Week 12	xx (xx)	xx (xx)	xx (xx to xx)	.xx
Week 24	xx (xx)	xx (xx)	xx (xx to xx)	.xx
Overall SZF effect				.xx
TG at	*n* = xx	*n* = xx		
Week 0	xx (xx)	xx (xx)	xx (xx to xx)	.xx
Week 12	xx (xx)	xx (xx)	xx (xx to xx)	.xx
Week 24	xx (xx)	xx (xx)	xx (xx to xx)	.xx
Overall SZF effect				.xx
LDL at	*n* = xx	*n* = xx		
Week 0	xx (xx)	xx (xx)	xx (xx to xx)	.xx
Week 12	xx (xx)	xx (xx)	xx (xx to xx)	.xx
Week 24	xx (xx)	xx (xx)	xx (xx to xx)	.xx
Overall SZF effect				.xx
HDL at	*n* = xx	*n* = xx		
Week 0	xx (xx)	xx (xx)	xx (xx to xx)	.xx
Week 12	xx (xx)	xx (xx)	xx (xx to xx)	.xx
Week 24	xx (xx)	xx (xx)	xx (xx to xx)	.xx
Overall SZF effect				.xx
HbA1c at	*n* = xx	*n* = xx		
Week 0	xx (xx)	xx (xx)	xx (xx to xx)	.xx
Week 12	xx (xx)	xx (xx)	xx (xx to xx)	.xx
Week 24	xx (xx)	xx (xx)	xx (xx to xx)	.xx
Overall SZF effect				.xx
BP at	*n* = xx	*n* = xx		
Week 0	xx (xx)	xx (xx)	xx (xx to xx)	.xx
Week 4	xx (xx)	xx (xx)	xx (xx to xx)	.xx
Week 8	xx (xx)	xx (xx)	xx (xx to xx)	.xx
Week 12	xx (xx)	xx (xx)	xx (xx to xx)	.xx
Week 16	xx (xx)	xx (xx)	xx (xx to xx)	.xx
Week 20	xx (xx)	xx (xx)	xx (xx to xx)	.xx
Week 24	xx (xx)	xx (xx)	xx (xx to xx)	.xx
Overall SZF effect				.xx
ACR at	*n* = xx	*n* = xx		
Week 0	xx (xx)	xx (xx)	xx (xx to xx)	.xx
Week 4	xx (xx)	xx (xx)	xx (xx to xx)	.xx
Week 8	xx (xx)	xx (xx)	xx (xx to xx)	.xx
Week 12	xx (xx)	xx (xx)	xx (xx to xx)	.xx
Week 16	xx (xx)	xx (xx)	xx (xx to xx)	.xx
Week 20	xx (xx)	xx (xx)	xx (xx to xx)	.xx
Week 24	xx (xx)	xx (xx)	xx (xx to xx)	.xx
Overall SZF effect				.xx
ADDQoL at	*n* = xx	*n* = xx		
Week 0	xx (xx)	xx (xx)	xx (xx to xx)	.xx
Week 12	xx (xx)	xx (xx)	xx (xx to xx)	.xx
Week 24	xx (xx)	xx (xx)	xx (xx to xx)	.xx
Overall SZF effect				.xx

^a^*p* values comparing between-group differences over the entire 24-week trial

The TCM symptom and total scores will be described from the baseline to 24 weeks after medication in both groups. These symptoms include physical and mental fatigue, dry mouth and throat, lower back weakness and pain, qi deficiency, listlessness, spontaneous perspiration, night sweats, dark purplish lips, edema, and dysphoria in the chest, palms, and soles.

The improvement of TCM syndromes will be evaluated according to the symptom score method, which is conducted according to the nimodipine method [15]. Efficacy index = (score before treatment − score after treatment) /score before treatment × 100%. The criteria were as follows:

(a) *Clinical control* is defined as the resolution of most symptoms after treatment (efficacy index ≥ 95%); (b) *obvious therapeutic effect* is defined as marked improvement in most clinical symptoms (efficacy index ≥70%); (c) *with therapeutic effect* is defined as experiencing symptomatic relief (efficacy index ≥30%); (d) *invalid*: no significant improvement or symptom exacerbation (efficacy index < 30%).

Clinical efficiency will be calculated using the formula: Clinical efficiency = (clinical control case number + obvious therapeutic effect case number + with therapeutic effect number)/total number of cases × 100%.

The clinical efficiency of TCM syndrome scores after 24 weeks of treatment will be calculated for both groups (Table 6).

**Table 6:** TCM syndrome score analysis

		SZF	SZF		Irbesartan	Irbesartan	
		Week 0	Week 24	***p*** Value	Week 0	Week 24	***p*** Value
		*n* = xx	*n* = xx		*n* = xx	*n* = xx	
Evaluation of TCM syndrome	Physical and mental fatigue	xx (xx)	xx (xx)	.xx	xx (xx)	xx (xx)	.xx
	Dry mouth and throat	xx (xx)	xx (xx)	.xx	xx (xx)	xx (xx)	.xx
	Weakness and soreness of the lower back and knees	xx (xx)	xx (xx)	.xx	xx (xx)	xx (xx)	.xx
	Qi deficiency and listlessness	xx (xx)	xx (xx)	.xx	xx (xx)	xx (xx)	.xx
	Spontaneous perspiration	xx (xx)	xx (xx)	.xx	xx (xx)	xx (xx)	.xx
	Night sweat	xx (xx)	xx (xx)	.xx	xx (xx)	xx (xx)	.xx
	Dysphoria in chest, palms, and soles	xx (xx)	xx (xx)	.xx	xx (xx)	xx (xx)	.xx
	Dark purplish lips	xx (xx)	xx (xx)	.xx	xx (xx)	xx (xx)	.xx
	Edema	xx (xx)	xx (xx)	.xx	xx (xx)	xx (xx)	.xx
Efficacy index	Clinical control	xx (xx%)	xx (xx%)	.xx	xx (xx%)	xx (xx%)	.xx
	Obvious therapeutic effect	xx (xx%)	xx (xx%)	.xx	xx (xx%)	xx (xx%)	.xx
	With therapeutic effect	xx (xx%)	xx (xx%)	.xx	xx (xx%)	xx (xx%)	.xx
	Invalid	xx (xx%)	xx (xx%)	.xx	xx (xx%)	xx (xx%)	.xx
	Overall clinical efficiency	xx (xx%)	xx (xx%)	.xx	xx (xx%)	xx (xx%)	.xx

### Safety analyses

The safety analysis will analyze all patients in the safety set. The laboratory index will be listed individually in the following situations: the conversion of normal to abnormal laboratory findings and the occurrence of abnormal hepatic and renal function during the treatment; additionally, the association between conversion and medications will be analyzed. The type, severity, occurrence, and relationship between the intervention drug and all adverse events experienced during treatment will be tabulated (Table 7). The number and frequency of adverse events will be calculated for both groups. The occurrence of severe adverse events and subsequent drug discontinuation will be recorded in detail.

**Table 7:** List of all adverse effects reported during the trial

Description	SZF, number	Irbesartan, number	Severity	Related to trial
xx	xx	xx	xx	xx
xx	xx	xx	xx	xx
xx	xx	xx	xx	xx

Any AE will be classified into three levels: mild, patients are able to tolerate discomfort without special treatment; moderate, patient will require special treatment to recover and tolerate the pain and discomfort; and severe, AEs that may lead to hospitalization, extended hospitalization, chronic severe disability, incapacity, or mortality. The relationship between AE and drugs will be assessed using standards from Measures for the Reporting and Monitoring of Adverse Drug Reactions, which was issued by the 2011 Ministry of Health of the People’s Republic of China.

The following relevance evaluations are listed in Table 8: (1) determining a possible relationship between the duration of drug use and suspicious adverse reactions; (2) the consistency of suspicious AE with other types of AE that are already known; (3) the explanations of the underlying mechanism of suspicious AE using pathological status, drug combination, and previous patient therapy; (4) the possibility of AE reduction or resolution after drug withdrawal or dosage reduction; and (5) the consistent occurrence of the same reaction after re-administration of the same drug. The incidence of adverse effects (AEs) will be compared between groups.

**Table 8:** Relevance evaluation between AE and drugs

Judgment results	Judgment index				
	1	2	3	4	5
**Definite relevance**	+	+	−	+	+
**Probable relevance**	+	+	−	+	?
**Difficult to determine**	+	+	±	±	?
**Probable irrelevance**	+	−	±	±	?
**Definite irrelevance**	−	−	+	-	-

+ means affirmative, − means negative, ± means difficult to determine, ? means unclear situation. The incidence of ADR is calculated by using the sum of the cases of 1 + 2 + 3 as numerator, and the number of all cases as denominator

The safety assessment will include routine blood, urine, and stool tests; electrocardiogram; liver function tests, including alanine aminotransferase, aspartate aminotransferase, alkaline phosphatase, gamma-glutamyl transferase, and serum total bilirubin; and kidney function tests including blood urea nitrogen, SCr, uric acid, and β2-microglobulin levels at baseline, week 12, and week 24 (Table 9). Safety analysis will describe the distribution by qualitative judgment, which counts the number of cases and their proportion of abnormal indexes by cross-tabulation.

**Table 9:** Safety assessment

		0 W			12 W			24 W		
		SZF	Irbesartan	***p*** value	SZF	Irbesartan	***p*** value	SZF	Irbesartan	***p*** value
		(***n*** = xx)	(***n*** = xx)		(***n*** = xx)	(***n*** = xx)		(***n*** = xx)	(***n*** = xx)	
**Hematology**	Red blood cell	xx (xx)	xx (xx)	.xx	xx (xx)	xx (xx)	.xx	xx (xx)	xx (xx)	.xx
	White blood cell	xx (xx)	xx (xx)	.xx	xx (xx)	xx (xx)	.xx	xx (xx)	xx (xx)	.xx
	Hemoglobin	xx (xx)	xx (xx)	.xx	xx (xx)	xx (xx)	.xx	xx (xx)	xx (xx)	.xx
	Platelet	xx (xx)	xx (xx)	.xx	xx (xx)	xx (xx)	.xx	xx (xx)	xx (xx)	.xx
**Urine test**	Glucose	xx (xx)	xx (xx)	.xx	xx (xx)	xx (xx)	.xx	xx (xx)	xx (xx)	.xx
	Ketone	xx (xx)	xx (xx)	.xx	xx (xx)	xx (xx)	.xx	xx (xx)	xx (xx)	.xx
	Occult blood	xx (xx)	xx (xx)	.xx	xx (xx)	xx (xx)	.xx	xx (xx)	xx (xx)	.xx
	Erythrocytes	xx (xx)	xx (xx)	.xx	xx (xx)	xx (xx)	.xx	xx (xx)	xx (xx)	.xx
	Leukocytes	xx (xx)	xx (xx)	.xx	xx (xx)	xx (xx)	.xx	xx (xx)	xx (xx)	.xx
**Stool routine**	Fecal leukocytes	xx (xx)	xx (xx)	.xx	xx (xx)	xx (xx)	.xx	xx (xx)	xx (xx)	.xx
	Feces occult blood	xx (xx)	xx (xx)	.xx	xx (xx)	xx (xx)	.xx	xx (xx)	xx (xx)	.xx
**Liver function**	ALT	xx (xx)	xx (xx)	.xx	xx (xx)	xx (xx)	.xx	xx (xx)	xx (xx)	.xx
	AST	xx (xx)	xx (xx)	.xx	xx (xx)	xx (xx)	.xx	xx (xx)	xx (xx)	.xx
	ALP	xx (xx)	xx (xx)	.xx	xx (xx)	xx (xx)	.xx	xx (xx)	xx (xx)	.xx
	GGT	xx (xx)	xx (xx)	.xx	xx (xx)	xx (xx)	.xx	xx (xx)	xx (xx)	.xx
	TBIL	xx (xx)	xx (xx)	.xx	xx (xx)	xx (xx)	.xx	xx (xx)	xx (xx)	.xx
**kidney function**	UA	xx (xx)	xx (xx)	.xx	xx (xx)	xx (xx)	.xx	xx (xx)	xx (xx)	.xx
	β2-microglobulin	xx (xx)	xx (xx)	.xx	xx (xx)	xx (xx)	.xx	xx (xx)	xx (xx)	.xx
	Serum albumin	xx (xx)	xx (xx)	.xx	xx (xx)	xx (xx)	.xx	xx (xx)	xx (xx)	.xx
	Blood urea nitrogen	xx (xx)	xx (xx)	.xx	xx (xx)	xx (xx)	.xx	xx (xx)	xx (xx)	.xx
**Electrocardiogram**		xx (xx%)	xx (xx%)	.xx	xx (xx%)	xx (xx%)	.xx	xx (xx%)	xx (xx%)	.xx
**Adverse events**	Mild	xx (xx%)	xx (xx%)	.xx	xx (xx%)	xx (xx%)	.xx	xx (xx%)	xx (xx%)	.xx
	Moderate	xx (xx%)	xx (xx%)	.xx	xx (xx%)	xx (xx%)	.xx	xx (xx%)	xx (xx%)	.xx
	Severe	xx (xx%)	xx (xx%)	.xx	xx (xx%)	xx (xx%)	.xx	xx (xx%)	xx (xx%)	.xx
	Overall AEs	xx (xx%)	xx (xx%)	.xx	xx (xx%)	xx (xx%)	.xx	xx (xx%)	xx (xx%)	.xx

### Imputation of missing data

Multiple imputations will be used for subjects with missing primary outcome data. Variables will be included in the multiple imputations, including the baseline 24-h UP, 24-h UP every 4 weeks, HbA1c, age, sex, and the characteristics and possible influencing factors of 24-h UP change. The missing data will be imputed five times. For missing secondary and safety outcomes, the results will be analyzed according to the actual data; the missing data will not be imputed nor will the model be adjusted. Furthermore, we will conduct a per-protocol analysis that excluded those with missing outcome data.

## Changes to the original SAP

The original SAP was created based on the initial study protocol and the related planned analyses. The focal point of the study protocol update and/or SAP revision since the first version, including the additional ACR measurement, the ADDQoL will be added as secondary outcomes, and the changes of outcome analysis. An interim analysis will be performed to evaluate measures of effectiveness and safety.

## Conclusions

The present study is a 24-week, randomized, multicentric, double-blinded, double-dummy clinical trial. Unbiased evaluation will be conducted on the clinical data in the trial to increase the credibility of the results and provide substantial evidence for further studies regarding the treatment of DKD.

## Status

The trial commenced in August 2014 and is scheduled to be completed in June 2022. Currently, 100 patients have been recruited.

## References to files

The data management plan and statistical master file were developed and maintained by a third-party statistical company. Trial master files were developed and retained by the Guang’anmen Hospital (principal investigators unit). The SOPs were developed collectively by the principal investigators and participating centers and were preserved separately.

## Supplementary Information

**Additional file 1.** Populated checklist.

## Data Availability

Not applicable. The trials discussed in this publication will be published or published separately. This project did not generate any data or material. Data sharing is not applicable to this article, as no datasets were generated or analyzed in this study.

## Abbreviations

DKD: Diabetic kidney disease
SZF: Shenzhou formula
CKD: Chronic kidney disease
ESRD: End-stage renal disease
RASS: Renin-Angiotensin-Aldosterone system
TCM: Traditional Chinese medicine
ARB: Angiotensin receptor blockade
GFR: Glomerular filtration rate
24 h-UP: 24-h urinary protein
SAP: Statistical analysis plan
CIs: Confidence intervals
FAS: Full analysis set
PPS: Per protocol set
SS: Safety set
FBG: Fasting blood glucose
ADDQoL: The audit of diabetes-dependent quality of life 19
AEs: Adverse effects
ACR: Albumin to creatinine ratio
